# Genetics of Darier’s Disease: New Insights into Pathogenic Mechanisms

**DOI:** 10.3390/genes16060619

**Published:** 2025-05-23

**Authors:** Barbara Moschella, Sabrina Busciglio, Enrico Ambrosini, Sofia Cesarini, Luca Caramanna, Sara Zanelli, Ilenia Rita Cannizzaro, Anita Luberto, Antonietta Taiani, Mirko Treccani, Erika De Sensi, Patrizia Caggiati, Cinzia Azzoni, Lorena Bottarelli, Bruno Lorusso, Costanza Anna Maria Lagrasta, Anna Montanaro, Luca Pagliaro, Raffaella Zamponi, Andrea Gherli, Davide Martorana, Michele Maria Dominici, Maria Beatrice De Felici Del Giudice, Paola Mozzoni, Enrico Maria Silini, Iria Neri, Claudio Feliciani, Giovanni Roti, Vera Uliana, Valeria Barili, Antonio Percesepe

**Affiliations:** 1 Medical Genetics, Department of Medicine and Surgery, University of Parma, 43126 Parma, Italy; 2 Medical Genetics, University Hospital of Parma, 43126 Parma, Italy; 3 Medical Genetics, IRCCS Azienda Ospedaliero-Universitaria di Bologna, 40138 Bologna, Italy; 4 Department of Food and Drug, University of Parma, 43126 Parma, Italy; 5 Pathology Unit, Department of Medicine and Surgery, University of Parma, 43126 Parma, Italy; 6 Translational Hematology and Chemogenomics (THEC), University of Parma, 43126 Parma, Italy; 7 Dermatology Unit, Department of Medicine and Surgery, University of Parma, 43126 Parma, Italy; 8 Department of Medicine and Surgery, University of Parma, 43126 Parma, Italy; 9 Dermatology Unit, IRCCS Azienda Ospedaliero-Universitaria di Bologna, 40138 Bologna, Italy

**Keywords:** Darier disease, *ATP2A2*, SERCA2 dysfunction and calcium homeostasis, UPR, skin disease, epidermal signaling pathways, therapeutic strategies

## Abstract

Darier′s disease (DD) is a rare, autosomal dominant genodermatosis caused by pathogenic variants in the ATP2A2 gene, which encodes the SERCA2 protein, an endoplasmic reticulum ATPase Ca^2+^ transporter. These mutations impair the intracellular calcium homeostasis leading to increased protein misfolding, endoplasmic reticulum (ER) stress response, and the activation of the unfolded protein response (UPR), culminating in keratinocyte apoptosis and anomalies in interfollicular epidermal stratification. Clinically, the disease is characterized by the presence of skin lesions with hyperkeratotic papules and an increased susceptibility to inflammatory reactions, bacterial and viral infections. The histological hallmarks include acantholysis, dyskeratosis, and increased apoptotic keratinocytes, referred to as “corp ronds”. The SERCA2b isoform is expressed not only in the epidermis but it is present ubiquitously in all tissues, suggesting that its alteration may have multi-organ effects. The review aims to provide a broad overview of the pathology, from intracellular dysfunction to the clinical manifestations, elucidating the molecular effects of SERCA2 variants found in DD patients and exploring the potential cell signaling pathways that may contribute to disease progression. Beginning with an examination of the cellular alterations, our work then shifts to exploring their impact in an organ-specific context, providing insights into new potential therapeutic strategies tailored to clinical manifestations.

## 1. Introduction

Darier’s disease (DD; MIM #124200), also known as follicular keratosis, is a rare genodermatosis characterized by the deregulation of the epidermal keratinization process. With an autosomal dominant inheritance, DD has an estimated prevalence ranging from 1:30,000 to 1:100,000 individuals, with no gender differences [1].

The onset of the disease generally occurs during adolescence or early adulthood, more rarely in childhood, and then follows a chronic-recurrent course in adulthood.

The typical disease manifestation consists of keratotic papules of varying sizes mainly located in seborrheic areas of the skin. These papules are often confluent, itchy, and may evolve into warty plaques with scabs [2].

Histological examination shows clear signs of dyskeratosis in the stratum corneum which is an abnormal keratinization occurring prematurely within individual cells or groups of cells below the stratum granulosum associated with keratotic plug formation and parakeratosis, which is characterized by the retention of nucleate cells in the stratum corneum, usually with no nuclei. The stratum spinosum, on the other hand, shows considerable thickening, with acantholysis, which refers to the separation of epidermal cells due to a loss of cell adhesions in the spinous layer, and abnormal keratinocytes, including characteristic round bodies and Darier’s granules, mainly located in the upper layers [3].

Skin lesions in patients with DD can be exacerbated by secondary cellular stressors, such as UVB irradiation, heat, secondary infections, and friction [2].

The gene responsible for DD is *ATP2A2*, located on chromosome 12q23–24.1, and was identified in 1999 by Hovnanian et al. [4]. Variants in *ATP2A2* correlate with an impaired function of the isoform b of the sarco/endoplasmic reticulum ATPase protein (SERCA2), a calcium pump distributed throughout the endoplasmic reticulum (ER).

The functional deregulation of SERCA2 activity leads to altered intracellular calcium (Ca^2+^) homeostasis and the post-translational misfolding of neosynthesized proteins, resulting in ER stress induction. Abnormal intracellular Ca^2+^ signaling appears to correlate with a loss of desmosomal junctions in basal cells with a subsequent acantholysis and cleavage of the epidermal suprabasal layer. This aberrant skin phenotype highlights the importance of the distinct regulation of the process of keratinization, which must be highly and properly preserved to maintain the protective function of the epidermal barrier, as the body’s first line of defense against the external environment.

Although the disease is associated with a clear disruption of the normal process of keratinization and multilayered stratification of the epidermis, the correlation between SERCA2b malfunction and the molecular mechanism triggering the manifestation of the disease is still unknown.

Our main objective is to provide a broad perspective on the molecular and systemic aspects involved in disease pathogenesis, discussing empirical findings from the literature and clinical therapeutic implications.

Therefore, this review offers in-depth analyses of the different cardiac, neurological, and metabolic manifestations that may occur with the typical skin manifestations of Darier’s disease, investigating all possible molecular pathways involved in the altered skin phenotype. In addition, we aim to highlight any existing gaps in current knowledge and propose further insights into research studies to better understand the pathophysiological mechanisms underlying the disease.

## 2. Molecular Pathogenesis

### 2.1. Darier’s Disease Skin Clinical Features

First described in 1889 by the French dermatologist Ferdinand-Jean Darier, Darier’s disease is a skin disorder caused by a disruption of the Ca^2+^ homeostasis at the epidermic level [1]. In the keratinocytes of DD patients, *ATP2A2* pathogenic variants affect the functional domains of the ATPase transporter SERCA2, impairing the activity of the protein, which is ubiquitously expressed, but predominantly in the epidermis [1].

The histopathologic features of DD include focal dyskeratosis characterized by acantholysis with cleavage between suprabasal keratinocytes due to the loss of desmosomal junctions, and hyperkeratosis in skin lesions. Dyskeratotic keratinocytes are cells that undergo premature differentiation and appear as rounded eosinophilic cells under the microscope. These keratinocytes are apoptotic and are termed “corps ronds” in the stratum spinosum and “corps grains” in the upper epidermal layer [5]. Lesions frequently affect intertriginous areas of the skin, where they can ulcerate and become infected. Palmoplantar keratoderma and onychopathy can also be observed and, in some female cases, symptoms worsen during the premenstrual period [3].

Darier’s disease can manifest through different clinical signs, which are classified into acral hemorrhagic, cornifying, hypertrophic, linear, and comedonal disease types [6]. Specifically, the acral hemorrhagic form presents with hemorrhagic vesicles on the palms and the dorsal fingers, which develop into black macules. This form is associated with *ATP2A2* mutations, which also affect blood vessels, as well as the skin. The hypertrophic type is characterized by warty lesions, mainly in the intertriginous areas. The horny clinical variant is a subcategory of the hypertrophic variant and typically presents with hypertrophic plaques on the legs, characterized by the presence of cutaneous horns. The comedonal variant manifests as acneiform eruptions on the head and neck, with clinical and histopathologic similarity to verrucous dyskeratomas.

Notably, a case report described a 42-year-old patient presenting with three coexisting forms: comedonal DD on the face, cornifying DD on the upper back, and hypertrophic DD on the legs. In addition to the classic histopathology of DD, multiple dyskeratoma verrucous-like structures in the comedonal type, compact hyperkeratosis in the cornifying type, and significant papillomatosis in the hypertrophic type were noted [7].

The somatic mosaicism of *ATP2A2* due to post-zygotic mutations is associated with acantholytic dyskeratotic nevi along the Blaschko lines, referred to as segmental Darier’s disease.

Clinically, the classical lesions are keratotic papules, frequently located on the trunk, and acral pits and wart-like lesions, mostly found on the hands [8]. Coexisting rare non-classical lesions may include acral keratoderma, leucodermic macules, giant comedones, keloid-like vegetations, and acral hemorrhagic blisters [8]. Nails can also be affected, displaying V-shaped notches at the free edge, longitudinal erythronychia (red discoloration of the nail) and leukonykia (partial or complete whitening of the nail plate), as well as ridging and splitting [9]. Genital involvement, especially on female genitalia, is more frequent in patients with severe disease. Lesions are present as hyperkeratotic papules, erosions, or whitish plaques, often localized on the labia majora, labia minora, and perineal region. The severity of genital manifestations correlates with the overall disease load. Genital lesions may be asymptomatic, or may cause pruritus, discomfort, or secondary infections, impacting the overall life. The identification of these manifestations is essential for proper management and differential diagnosis from other genital dermatoses [10]. Oral mucosal involvement frequently shows “cobblestone-like” papules and leukokeratosis, occurring in approximately 15% of patients. Additionally, some patients may experience gingival hyperplasia, macroglossia, xerostomia, and recurrent parotid gland obstruction [9]. DD skin lesions can also develop on eyelids, with additional ophthalmic complications including corneal epithelial ulceration, conjunctival keratosis, conjunctival hyperemia, blepharitis, and dryness [11,12].

Cutaneous lesions are often accompanied by itching, burning sensations, and pain [13]. Furthermore, when intertriginous areas are affected, the lesions may have an unpleasant odor, contributing to social isolation [14].

Several DD variants associated with non-classical DD cutaneous manifestations have been reported [8]. The bullous variant is distinguished by the presence of vesicles and blisters, triggered by sweating, stress, or fever [15]. The hemorrhagic variant is characterized by superficial hemorrhages in acral areas, induced by mechanical stress [16]. The variant with prominent comedones primarily affects the face, presenting no other clinical signs [17]. Impetigination and eczematization are frequently observed, and DD individuals exhibit an increased susceptibility to infections, particularly by Staphylococcus aureus and Herpes simplex [18]. The most relevant extracutaneous manifestations are neuropsychiatric, including seizures, learning disabilities, mild intellectual disability, schizophrenia, and bipolar disorder [19].

### 2.2. Genetics of Darier’s Disease

Darier’s disease displays autosomal dominant inheritance and often occurs within families. The incidence of sporadic cases is reported as approximately 40–50%, with a high penetrance of greater than 95%.

**Figure 1:**
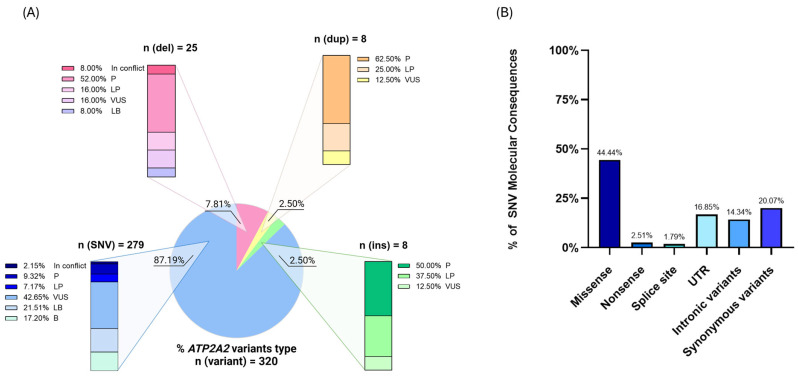
Classification of *ATP2A2* variants indexed in the ClinVar database. (**A**) The pie chart represents the distribution of 320 reported variants based on their type: single nucleotide variants (SNVs, 87.19%), deletions (del, 7.81%), insertions (ins, 2.50%), and duplications (dup, 2.50%). The bar charts detail the clinical significance of each variant type according to the American College of Medical Genetics (ACMG) criteria: P (Pathogenic), LP (Likely Pathogenic), VUS (Variant of Uncertain Significance), LB (Likely Benign), B (Benign), and In Conflict (conflicting interpretations among submitters). SNVs account for the majority of variants, with a high proportion classified as VUS (42.65%). Conversely, deletions, insertions, and duplications show a greater fraction of P or LP variants. Data were accessed from ClinVar on 28 March 2025. (**B**) The bar graph illustrates the distribution of SNVs according to their molecular consequences, with a predominant presence of missense variants. UTR = untranslated region.

By investigating genetic variants, a total of 320 alterations are reported in the ClinVar database [20] [accessed on 28 March 2025], of which only 202 are classified as pathogenicity class 3, 4, or 5 according to ACMG criteria (as illustrated in Figure 1A) [21]. About 60% of these 202 variants are described as Variants of Unknown Significance (VUS): this group is entirely composed of missense variants and alterations of the untranslated regions (UTRs). Accordingly, missense changes are also the most represented among the Pathogenic (P) or Likely Pathogenic (LP) variants in ClinVar, accounting for nearly one-third of these variants. The loss-of-function is a common pathogenic mechanism (pLI = 1 in gnomAD) [22], and the other reported P/LP variants are, in order of frequency, frameshift, nonsense, and splice-site molecular changes (Figure 1B).

**Figure 2:**
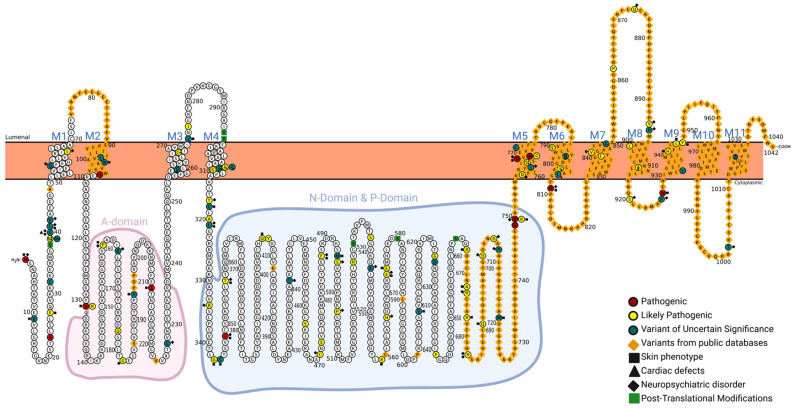
Schematic representation of the SERCA2b protein topology encoded by the human ATP2A2 gene. The diagram highlights the 11 transmembrane domains (M1–M11) of the SERCA2 protein, with domains M1–M10 forming the core structure and M11 being specific for the SERCA2b isoform, which corresponds to the extended C-terminal helix. The key cytoplasmic functional regions—namely, the actuator domain (A-domain, in pink), and the nucleotide-binding and phosphorylation domains (N-domain and P-domain, in light blue)—are annotated according to their respective amino acid boundaries. Individual amino acid residues are displayed, and annotated sites include post-translational modifications and functionally relevant residues based on existing biochemical and structural data. The distribution of variants across functional domains are derived from the UniProt and Pfam databases, integrating experimental findings and computational predictions.

Mutation sites are distributed throughout genes with no particular hotspots within the various protein domains (Figure 2).

In order to examine other reported variants and connect them with a clinical phenotype, we expanded the search to the literature. We screened a total of 112 articles. We identified a total of 189 patients with variants in the ATP2A2 gene and a diagnosis of Darier’s disease; of these, 92 were female, 64 were male, and for 33 the sex was not reported (Table 1 and Supplementary Table S1 [23,24,25,26,27,28,29,30,31,32,33]).

**Figure 3:**
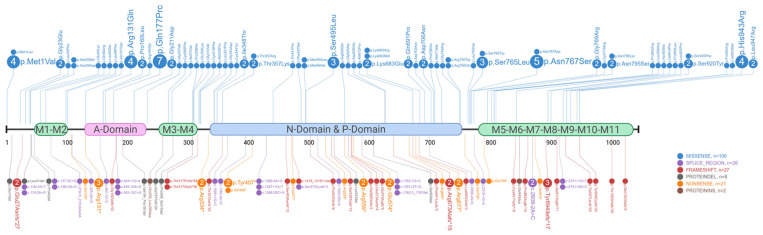
Lollipop diagram depicting SERCA2 literature-based variants. The numbers within circles correspond to the recurrence of the variants described in Supplementary Table S1 and the colors are indicative of the corresponding molecular type: missense (in blue, n = 106), frameshift (in red, n = 27), nonsense (in orange, n = 21), splicing (in purple, n = 26), protein insertion (in brown, n = 2), and protein deletion (in grey, n = 8). Functional domains are depicted: the transmembrane domains (M1–M11, in green), the actuator domain (A-domain, in pink), and the phosphorylation/nucleotide-binding domains (P- and N-domains, in blue).

A dermatological phenotype was reported in almost all patients, from mild (30%) to moderate (44%) or severe (26%). Approximately one-third (34%) of the described patients also had neuropsychiatric disorders, ranging from learning disabilities to major depression. Only three patients were reported with cardiological manifestations (mostly non-specific EKG alterations). The total number of different variants identified is 143, since some of the patients are related and/or harbor the same variant (Figure 3). These include point nucleotide substitutions and missense, nonsense, and splicing variants with the loss or gain of a splice site, as well as deletions and insertions. Importantly, most patients considered carry missense variants (75%), as highlighted in Figure 3.

All of the variants are distributed along the entire primary sequence of *ATP2A2* and do not display any clusters within potential “hot-spot” regions. Many variants fall within the conserved sites of the three functional cytoplasmic domains of the protein, explaining the impairment of its functionality. Specifically, 34 clinical cases show variants located in the phosphorylation (P) domain, 32 in the nucleotide ATP-binding (N) domain, and 30 in the actuator (A) domain. Genetic variants affecting the protein start codon (e.g., p.Met1Val and p.Met1Leu) and leading to a complete loss of its synthesis, have been found in five different patients (Figure 3) [30,32,34].

A genotype–phenotype analysis in relation to the disease severity revealed no clear correlation, and therefore the classification of the disease severity still relies on clinical criteria.

However, Amar et al. recently published the first severity score, specifically tailored to DD, that can be useful in guiding treatment choices as well as assessing and documenting treatment response [35]. Specifically, the DD score is based on three different values weighted by correction factors. The first parameter is the severity score/objective score (A or oDD) which is calculated on the severity of specific symptoms, including the hyperkeratosis/induration of papules and erosions, each ranging from zero (none) to three (severe), and on the affected areas, such as axilla, inguinal, submammary, facial, and other regions, also ranging from zero to three. The second parameter, defined as the Extent (B) component, measures the percentage of the body surface area affected by DD lesions, with specific percentages assigned to different body regions (e.g., 4.5% for each arm and 18% for the trunk). The third and last parameter, the subjective DD (sDD or C) score, evaluates pain and itchiness, both ranging from zero (none) to ten (unbearable). The overall DD score is calculated using the formula: DD Score = A/5 + 7B/2 + C. The maximum possible DD score is 103, indicating the highest level of disease severity. It is based on the SCORAD (Scoring Atopic Dermatitis) concept and aims to facilitate disease management and treatment evaluation, although it does not include aspects of multi-organ disease.

In addition, DD patients carrying the same pathogenic variant show different clinical manifestations, highlighting the absence of a distinctive genotype–phenotype correlation. For example, the study by Amichai et al. [25] describes three related patients affected by DD harboring the same LP variant c.530A>C (p.Gln177Pro) and different clinical manifestations. The mother showed a moderate phenotype, while one of her sons suffered from severe disease with warty, greasy, hyperkeratotic papules and plaques over the scalp, upper trunk, and forearms. In contrast, the youngest son showed a mild form, with few skin and oral lesions. This phenotypic expressivity/variability may be partially explained by the influence of modifier genes, epigenetic factors, environmental triggers or skin microbiota subtype, which could mitigate the disease severity and account for individuals, also within the same family, which manifest very mild or nearly asymptomatic manifestations.

At the mechanistic level, the suggested etiology is haploinsufficiency, although this hypothesis remains unverified, as mice with a heterozygous inactivation of the SERCA2-encoding gene do not develop the corresponding human disease phenotypes, leaving the molecular mechanisms underlying these dominant disorders unresolved [36]. Indeed, some of the missense variants lead to disease symptoms which are more severe than those observed in patients with nonsense changes leading to the introduction of stop codons that eliminate the protein expression resulting in a severe loss of function [37]. Thereby, the stronger outcome for missense mutations may result from a likely gain-of-function mechanism. This interpretation is supported by two studies. The first generated mutant flies with alterations in multiple SERCA2 protein regions showing that the resulting phenotypes were possibly driven by a gain-of-function mechanism due to ionic leakage through the altered pumps. The second study showed that an overexpression of mutant SERCA2 leads to reduced solubility, protein aggregation, and increased polyubiquitinylated. Notably, mutant SERCA2 triggered keratinocyte proliferation (dyskeratosis) and apoptosis (acantholysis) [38].

Thereby, despite the identification of numerous *ATP2A2* variants, the lack of a clear genotype–phenotype correlation and the unresolved pathogenic mechanism highlights the need for mechanistic insights, prompting a closer examination of how SERCA2 dysfunction alters calcium homeostasis and cellular physiology in the epidermis and in the other organs.

### 2.3. Cellular Pathophysiology of the Skin Barrier

The ATPase transporter SERCA2 plays a fundamental role in maintaining intracellular calcium homeostasis by actively translocating two Ca^2+^ ions from the cytosol to the lumen of the ER, where Ca^2+^ is stored at higher concentrations. The catalytic cycle of the ATPase pump occurs via conformational changes between phases with high and low affinity for Ca^2+^, corresponding to the high- or low-energy phosphorylated states [39].

In the epidermis, most of the free Ca^2+^ originates from intracellular stores, such as the Golgi apparatus and the ER [1]. The replenishment of these calcium reserves is essential for maintaining calcium-dependent signaling processes crucial for epidermal homeostasis and response to extracellular stimuli.

**Figure 4:**
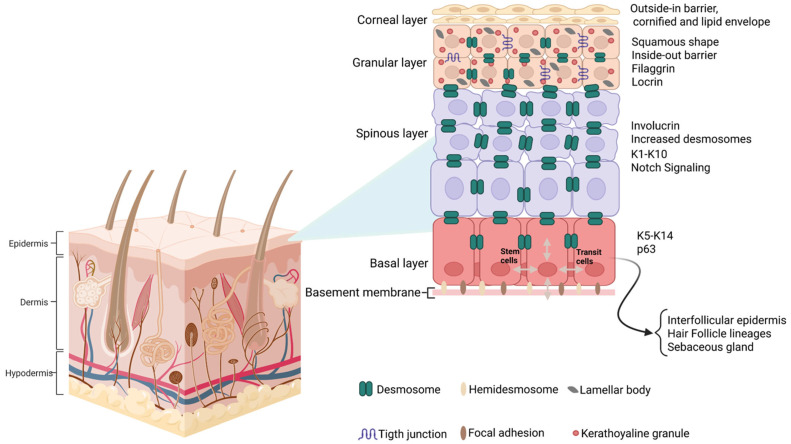
Illustration of the layers of the interfollicular epidermis and their specific keratinocyte differentiation markers. K1–K10 and K5–K14 represent keratin specific markers.

The continuous renewal of the epidermis during adult life occurs through the activity of the basal stem cells, which are a subpopulation of keratinocytes found in the basal layer, adjacent to the dermis, that give rise to differentiating cells in the suprabasal layers of the interfollicular epidermis (IFE), hair follicles, and sebaceous glands. Basal progenitor daughter cells are known as transit-amplifying cells (TACs) that can divide a limited number of times before committed terminal differentiation (Figure 4) [40]. Cell adhesion plays a key role in regulating the proper timing of cell differentiation and the progressive migration to the corneum stratum. The adhesion between basal cells and the extracellular matrix of the underlying basal lamina is mediated by hemidesmosomes, integrins (α6-β4), adherens junctions (E-cadherin), and desmosomes (desmoglein 3) [41].

The dyskeratotic and hyperkeratotic phenotype of DD is well known, but the underlying molecular mechanisms remain not fully understood. Alterations underneath these distinctive skin lesions could therefore be investigated in DD patients.

**Figure 5:**
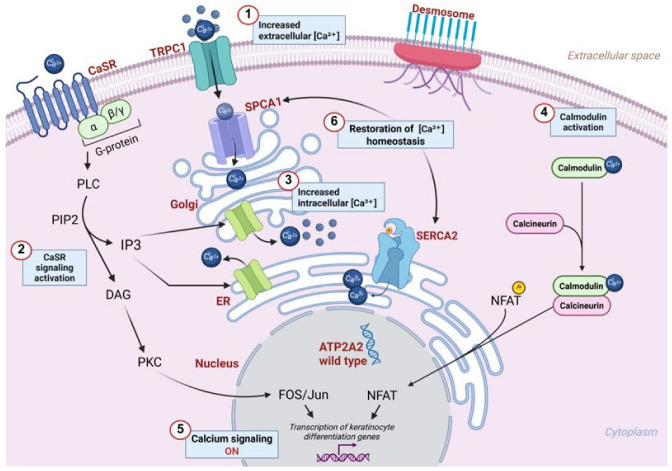
Calcium signaling regulation in keratinocytes. (**1**) Increased intracellular calcium levels are regulated by calcium influx through transient receptor potential cation channel subfamily C member 1 (TRPC1) and calcium transport into the Golgi apparatus via secretory pathway calcium ATPase 1 (SPCA1). Additionally, calcium is sequestered back into the endoplasmic reticulum by SERCA2, preventing cytosolic calcium overload. (**2**) Extracellular Ca^2+^ also represents a signal for activation of the G-protein-coupled calcium-sensing receptor (CaSR), which, via an intracellular signal transduction mechanism, actives phospholipase C (PLC). PLC hydrolyzes phosphatidylinositol 4,5-bisphosphate (PIP2) into the second messenger inositol 1,4,5-trisphosphate (IP3) and diacylglycerol (DAG). (**3**) IP3 binds to Ca^2+^ channels located on the ER and Golgi membrane, resulting in their opening and the release of Ca^2+^ from intracellular reserves into the cytosol. (**4**) Increased intracellular Ca^2+^ leads to the activation of a series of calcium-dependent proteins. One example is calmodulin, a Ca^2+^-binding protein, which in turn activates calcineurin phosphatase. (**5**) The latter mediates the dephosphorylation of nuclear factor of activated T-cells (NFAT), a transcription factor that migrates into the nucleus and activates gene expression typical of the Ca^2+^ signaling pathway. DAG leads to the activation of protein kinase C (PKC) that is involved in the phosphorylation and activation of intracellular effector proteins, including the Fos/Jun transcription factor family, mediating the transcription of keratinocyte differentiation genes. (**6**) Finally, calcium homeostasis is restored. This is accomplished by SERCA2 and SPCA1 transporters that mediate the reuptake of Ca^2+^ into intracellular stores in the ER and Golgi, respectively.

In the epidermis, calcium signaling organizes the keratinocyte differentiation processes. Under normal conditions, extracellular Ca^2+^ in the epidermis follows a gradient that progressively increases from the basal layer to the granulosum layer, and then drastically declines in the stratum corneum [42]. In the suprabasal epidermis, calcium is required for the formation of desmosomal junctions, which are absent in the basal layer, as they are responsible for inhibiting cell proliferation to initiate the keratinization process in the upper layers. The extracellular Ca^2+^ gradient in the IFE regulates the proper timing of cell differentiation through fine-tuned gene expression specific to each epidermal layer. An increase in the extracellular Ca^2+^ activates the calcium-sensing receptor (CaSR), triggering intracellular signal transduction inositol trisphosphate (IP3), which mediates Ca^2+^ release from ER and Golgi stores (Figure 5). Ca^2+^ propagation activates calcium-binding proteins such as calmodulin, which stimulates calcineurin phosphatase. The latter mediates the dephosphorylation of nuclear factor of activated T-cells (NFAT), enabling the nuclear translocation and activation of calcium-responsive genes [1].

Activation of CaSR in response to increased extracellular calcium leads to the release of the second messenger diacylglycerol (DAG), which subsequently activates different intracellular effector proteins, including the Fos/Jun transcription factor family (Figure 5). These transcription factors mediate the effects of Ca^2+^ on keratinocyte differentiation by promoting the sequential gene expression of differentiation markers such as K1, K10, involucrin, loricrin, and filaggrin [43].

To restore Ca^2+^ homeostasis, SERCA2 transporters reuptake Ca^2+^ into the ER and Golgi [1]. The balance between SERCA2 activity, intracellular signaling, and epidermal differentiation guides epidermal barrier maintenance (Figure 5).

Upon transitioning to the spinous layer, TACs silence basal cell marker keratins 5 and 14 (K5 and K14), downregulating the p63 signaling pathway (a member of the p53 family), and activating the NOTCH1 receptor-mediated signaling pathway, which promotes the arrest of cell proliferation and induces the expression of spinous-layer terminal differentiation markers, including Keratin 1 (K1) and Keratin 2 (K2), associated with the expression of the *Hes1* transcription factor [41] (as illustrated in Figure 4).

The progression of TACs into the granular layer is mediated by the expression of the epidermal differentiation complex (EDC), a genomic cluster composed of 50 genes essential for barrier formation and encoding for pro-filaggrin, loricrin, involucrin, and other proteins.

In addition, late spinous and granular cells begin to produce lamellar bodies, which store lipid components crucial for the assembly of the cornified envelope [41].

Although the disease is associated with a clear disruption of the normal process of keratinization and multiple stratifications of the epidermis, the correlation between SERCA2b impairment and the molecular mechanism triggering the manifestation of the disease is still unknown.

**Figure 6:**
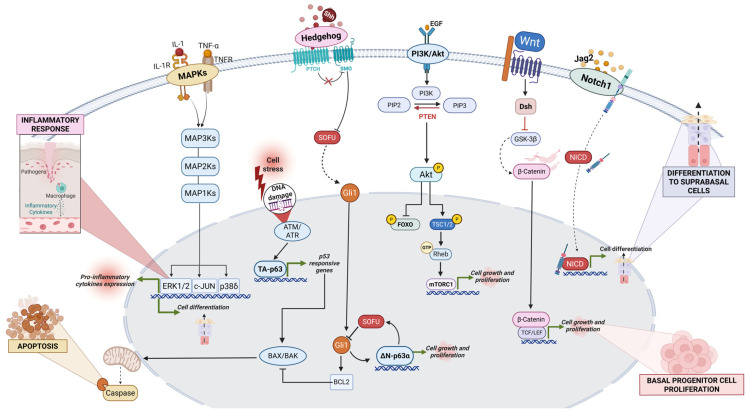
Overview of the signaling pathways regulating epidermal homeostasis. Fundamental signaling cascades involved in keratinocyte proliferation, differentiation, apoptosis, and response to stress or inflammation are depicted. ∆Np63α promotes basal progenitor proliferation, while NOTCH1 signaling drives their differentiation into suprabasal cells. The Sonic Hedgehog (SHH) pathway guides proliferation via induction of ∆Np63α and Bcl-2. PI3K/AKT signaling controls growth and proliferation through mTOR activation and FOXO inhibition. Wnt signaling maintains basal stem cell renewal and hemidesmosome organization. Inflammatory and stress responses activate MAPK cascades, leading to cytokine expression, differentiation, or apoptosis.

*Multiple signaling pathways* are essential in regulating epidermal homeostasis and function (as illustrated in Figure 6).

Epidermal homeostasis, as mentioned above, is tightly regulated by the expression of p63. In particular, the isoform ΔNp63α acts in the adult skin by preserving basal stem cell proliferative potential, while the isoform TAp63γ is primary expressed during embryonic development [44]. ΔNp63α is highly expressed in basal keratinocytes for the continuous renewal of senescent keratinocytes in the stratum corneum and for wound healing [45].

The **NOTCH1 receptor** signaling pathway finely regulates their transition to mature keratinocytes, playing an antagonistic role to p63 by inducing a switch from a proliferative to a differentiated phenotype through the expression of specific transcription factors (e.g., *Hes1*) [46].

The **Sonic Hedgehog** (SHH) pathway promotes basal cell proliferation in synergy with ΔNp63α [47] and plays an important role in hair follicle development, stimulating follicle downgrowth into the dermis [48].

In IFE, SHH activation promotes the expression of cell proliferation target genes, including ∆Np63α and the anti-apoptotic protein Bcl-2 [47], which is restricted to undifferentiated basal keratinocytes [49]. In contrast, suprabasal keratinocytes lose Bcl-2 expression, which is accompanied by a downregulation of the SHH pathway.

In addition, the **Wnt pathway** maintains IFE stem cells through an autocrine signaling mechanism. Basal stem cells induce the proliferation of adjacent cells by releasing Wnt ligands. In contrast, Axin2, a Wnt/β-catenin target gene, inhibits Wnt signaling via negative feedback, preventing uncontrolled basal proliferation [50].

Additionally, Wnt/β-catenin signaling regulates hemidesmosome adhesion complexes (HDs) in keratinocytes by reducing plectin and type XVII collagen expression in basal cells [51].

Moreover, in the skin, the mitogen-activated protein kinase (MAPK) families are critical regulators of various epidermal processes, including proliferation, differentiation and apoptosis, ascertaining proper epidermis homeostasis and response to environmental stimuli. Indeed, these signaling pathways combine extracellular signals and intracellular responses that are essential for maintaining skin integrity, barrier function, and wound healing. For instance, the **p38 MAPK** pathway specifically regulates the stress response to external stimuli and inflammation in the epidermis [52,53].

The **ERK1/2 MAPK** pathway is associated with cell proliferation and survival. It is activated in response to growth factors and mitogens, promoting keratinocyte proliferation during epidermal renewal and re-epithelialization following injury [52].

The **JNK signaling** pathway is largely involved in cellular stress responses and apoptosis, playing a role in keratinocyte differentiation and programmed cell death. It is particularly responsive to oxidative stress, UV radiation, and pro-inflammatory signals, thereby influencing skin aging and photodamage. Persistent JNK activation has been linked to inflammatory skin disorders and contributes to epidermal barrier defects [54].

Overall, the MAPK signaling network acts as a critical point in skin physiology, dynamically regulating keratinocyte fate decisions in response to intrinsic and extrinsic cues. The precise balance between these pathways determines whether cells proliferate, differentiate, or undergo apoptosis, ensuring epidermal homeostasis while also shaping the skin’s response to injury, infection, and disease.

The **phosphatidylinositol-3-kinase (PI3K)/protein kinase B (AKT)**-mediated pathway is fundamental for skin development, homeostasis, and repair, orchestrating keratinocyte proliferation, differentiation, survival, and barrier formation. This signaling is triggered by growth factor receptors (GFRs) [55], including epidermal growth factor receptor (EGFR), insulin-like growth factor receptor (IGFR), and fibroblast growth factor receptor (FGFR), in response to extracellular stimuli. In keratinocyte proliferation, the PI3K/AKT pathway boosts cell cycle progression by activating cyclin-dependent kinases (CDKs) and inhibiting apoptotic regulators such as BAD and FOXO transcription factors [56], thus ensuring the continuous renewal of the basal layer progenitors of the epidermis.

Moreover, PI3K/AKT modulates keratinocyte differentiation by controlling expression of involucrin, loricrin, and filaggrin markers, through the interaction with MAPK and NOTCH signaling to fine-tune epidermal maturation. For epidermal maintenance, PI3K/AKT cross-talks with the other pathways, like mTOR [56], and Wnt/β-catenin to promote cellular response to injury. Thereby, the PI3K/AKT pathway represents a central regulator of skin physiology, integrating external signals to maintain epidermal renewal, differentiation, and response to injury, while its dysregulation contributes to pathological conditions affecting skin integrity and function.

### 2.4. Skin Barrier Dysfunction Due to SERCA2 Alterations

Skin impairment caused by the SERCA2b malfunction highlights the key role that calcium plays in the regulation of cell adhesion and the process of keratinocyte terminal differentiation [1].

In the DD-affected epidermis, SERCA2b dysfunction disrupts the proper calcium signaling as exhibited by the presence of dyskeratotic lesions, with abnormal keratinization as a feature of the disease [57]. The formation and maintenance of desmosomes, crucial for epidermal integrity, are compromised, leading to desmosomal loss and acantholysis as observed in DD. Despite the presence of compensatory mechanisms, such as calcium influx and calcium transport into the Golgi via the secretory pathway, these pathways are insufficient to fully counteract the effects of SERCA2 dysfunction. The dyskeratotic and hyperkeratotic phenotype of DD is well known, but the underlying molecular mechanisms remain not fully understood. Alterations in these pathways could therefore be investigated in DD patients.

**Figure 7:**
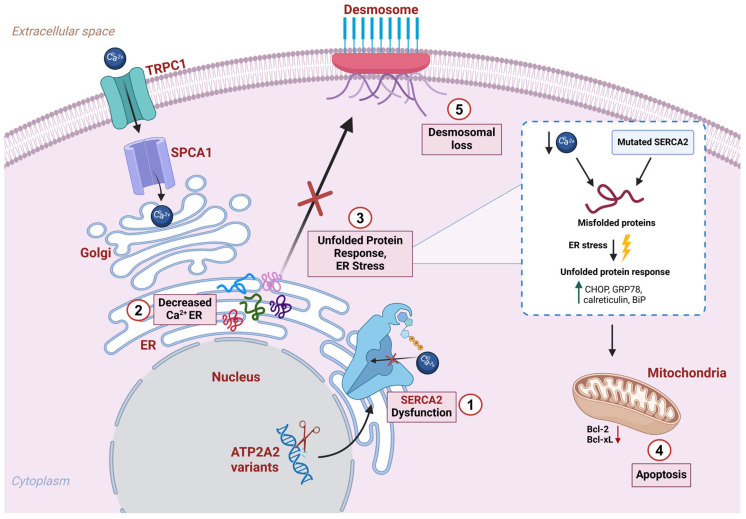
Proposed pathogenic mechanism associated with SERCA2 dysfunction. (**1**) *ATP2A2* variants impair the function of the SERCA2b pump, a protein responsible for transporting calcium ions into the endoplasmic reticulum (ER). This leads to disrupted intracellular calcium homeostasis. (**2**) The decrease in calcium reserves within the ER compromises essential cellular processes, particularly protein folding. As a result, misfolded proteins accumulate in the ER, triggering a protective cellular mechanism known as the unfolded protein response (UPR). (**3**) The UPR involves the upregulation of molecular chaperones, including CHOP, GRP78, calreticulin, and BiP, which attempt to refold or degrade the misfolded proteins and restore normal ER function. (**4**) Although cells attempt to compensate by enhancing calcium influx through channels such as TRPC1, and by transporting calcium into the Golgi apparatus via SPCA1 pumps, these mechanisms are insufficient to fully restore calcium homeostasis. If ER stress becomes prolonged or severe, it activates the mitochondrial apoptotic pathway, through the deregulation of Bcl-2 family proteins, including Bcl-2 and Bcl-xL, which play a key role in suppressing cell death under physiological conditions. (**5**) The altered calcium homeostasis and ER stress also has a profound impact on keratinocyte adhesion. This results in the disruption of cell–cell junctions (desmosomes) and a consequent loss of cohesion among epidermal cells (acantholysis), a hallmark feature of Darier’s Disease.

In addition, altered calcium homeostasis also affects intracellular signaling leading to desmosomal protein assembly defects and increased protein misfolding at the ER level (Figure 7).

Histologically, the disruption of desmosome–keratin complexes [5] has been observed along with the loss of cell adhesion, hyperproliferation of basal progenitor cells, and thinning of the spinous and granular layers.

One study reveals that SERCA2 mutated keratinocytes showed a marked reduction in mRNA levels encoding intermediate filament cytoskeleton proteins KRT1 and KRT10, as well as the desmosomal cadherin DSG1 (Figure 7), which are typically expressed in a differentiation-dependent manner in the suprabasal epidermis. Conversely, SERCA2 dysfunction leads to a modest effect on the expression of the primary keratin of the basal layer of the epidermis (KRT14) [58].

Moreover, the calcium level regulates Ca^2^-dependent chaperone proteins localized within the ER, which drive the folding process of neo-synthesized proteins. SERCA2 malfunction results in additional signaling consequences such as increased protein misfolding and the formation of protein aggresome in the ER (Figure 7) [2,38].

Indeed, the accumulation of these protein aggregates is the primary trigger of an ER stress condition, in response to which the cell activates an intracellular signaling pathway called the “unfolded protein response” (UPR) [59].

The activation of the UPR pathway is mediated by three transmembrane receptors: PERK, ATF6, and IRE1. Under stress conditions, when ER Ca^2+^ levels are reduced and misfolded proteins accumulate, GRP78 dissociates from the receptors, triggering the UPR pathway. Under stress conditions, when ER Ca^2+^ levels are reduced and misfolded proteins accumulate, GRP78 dissociates from the receptors, triggering the UPR pathway [60].

The UPR response leads to an increase in the synthesis of molecular chaperones while downregulating general translation [5].

Notably, SERCA2b protein expression is also greatly stimulated by the ER stress response to meet the increased Ca^2+^ demand. In fact, the ATP2A2 gene promoter contains conserved ER stress response elements (ERSE and UPRE) which mediate the expression of ER stress proteins [61,62].

In DD patients, carrying one functional copy of *ATP2A2*, an enhanced expression of SERCA2b is not accompanied by an efficient increase in its functional activity. Consequently, epidermal DD cells would not be able to restore homeostasis and cannot arrest the UPR response [5].

If ER stress persists, as in the pathological context of Darier’s disease, a cellular response shifts from adaptation to apoptosis, through the expression of the pro-apoptotic factor CHOP (Figure 7) [63]. Studies have shown that DD skin lesions display an enhanced expression of ER stress markers, such as CHOP, GRP78, calreticulin, and BiP, suggesting that the keratinocyte apoptosis in DD is mediated by the mitochondrial apoptosis pathway (Figure 7) [64].

However, another study suggests that DD keratinocytes may undergo apoptosis through a mitochondria-independent mechanism. In fact, this study observed an increased expression of poly (ADP-ribose) polymerase (PARP) and caspase 2 in DD keratinocytes, while the levels of the pro-apoptotic proteins BAK and BAX remained unchanged [5].

Other signaling pathway alterations have been investigated in DD patients, such as ERK hyper-activation in skin biopsies from DD patients compared to healthy individuals, supporting the hypothesis that MAPK pathway activation via ERK may be a result of SERCA2b dysfunction, highlighting it as a potential pathogenic driver in DD [58].

Darier’s disease is commonly misdiagnosed as eczema, seborrheic dermatitis, Hailey–Hailey disease (HHD), or acanthosis nigricans. Interestingly, HHD is associated with pathogenic variants in *ATP2C1,* which encodes the secretory pathway Ca^2+^-ATPase 1 (SPCA1) which is a calcium pump transporting Ca^2+^ and Mn^2+^ ions into the Golgi apparatus, where they are stored until needed. Although both DD and HHD involve abnormalities in calcium pumps and may share certain histologic similarities, their clinical and molecular characteristics are unique [65]. The haploinsufficiency of SPCA1 results in reduced Ca^2+^ sequestration in the Golgi, and impaired N-linked glycosylation, protein trafficking, and desmosome assembly. These dysfunctions underlie the partial suprabasal acantholysis characteristic of HHD, particularly affecting the spinous layer, which appears as a “dilapidated brick wall” [66]. In addition, as a phenotype feature, patients typically present oozing and sometimes erosive erythematous plaques in intertriginous areas such as the axillae, groin, and inframammary folds [67,68]. Further evidence of SPCA1’s critical role in Golgi function comes from studies showing its specific localization at the lateral rims of Golgi stacks and in the tubular regions of the trans-Golgi network. Together, these findings support the notion that SPCA1 dysfunction contributes to not only keratinocyte adhesion defects in HHD but also broader disturbances in membrane trafficking and calcium-dependent cellular processes [69]. In contrast, the dysregulation of ER calcium homeostasis in DD impairs protein folding and triggers ER stress and the unfolded protein response (UPR), ultimately leading to keratinocyte apoptosis. Histologically, this manifests as suprabasal acantholysis with the presence of apoptotic “corps ronds” which is a key diagnostic hallmark of DD [5]. In DD keratinocytes, anti-apoptotic signaling and increased proliferative capacity appear to be associated with TRPC1 overexpression, contributing to hyperkeratosis [70]. Increased TRPC1 expression also significantly enhances Ca^2+^ influx and the levels of anti-apoptotic protein such as Bcl-xL. Taken together, these findings suggest a novel role for TRPC1 in keratinocyte survival and a possible contribution to the skin cell pathologies associated with DD. In conclusion, the distinct localization and function of SPCA1 and SERCA2 reveal different pathomechanisms underlying HHD and DD. While SERCA2 regulates calcium levels in the endoplasmic reticulum (ER), affecting protein folding and cell survival pathways, SPCA1 maintains calcium homeostasis in the Golgi apparatus. This affects not only protein processing and desmosome assembly but also the structural integrity of the Golgi and vesicular trafficking. These differences result in two distinct clinical and histological diseases.

Moreover, studies have explored a possible association between DD and psoriasis, as both diseases share a similar pathogenetic mechanism, including abnormal cellular Ca^2+^ metabolism, ER stress, and protein misfolding (UPR). A peculiar difference is observed, indeed, in the expression of TRPC1 transporter, which is decreased in psoriasis and increased in DD [71]. Both diseases also display abnormal keratinocyte differentiation and disrupted epidermal stratification. One study showed that in DD, the acanthotic epidermis exhibits diffuse involucrin localization throughout the stratum spinosum and stratum granulosum, whereas in normal epidermis, involucrin is restricted to the cell membrane of the upper stratum spinosum and stratum granulosum keratinocytes [72]. Abnormal involucrin expression has also been found in psoriasis. However, cases of patients suffering with both DD and psoriasis have rarely been reported, and further studies are needed to clarify their relationship [71].

In DD, epidermal barrier disruption in skin lesions involves a significant upregulation of pathways related to epidermal repair, inflammation, and immune defense; hence, chronic skin inflammation represents a real DD hallmark. One study found Th17 immune cells in the dermal infiltrate of inflamed skin [9], along with an increased expression of the IL-17 cytokine. Notably, blocking the IL-23/IL-17 axis leads to an amelioration of the skin manifestations [73]. Although skin inflammation in DD results from epidermal barrier dysfunction and skin microbiome dysbiosis, mutations in *ATP2A2* may also impact immune function. One study showed that a loss of SERCA2 in B-cell precursors impairs their V(D)J recombination and subsequent maturation, reducing the number of mature B lymphocytes in some DD patients [74]. In addition, DD skin lesions displayed a reduced number of epidermal CD1a^+^ Langerhans cells and dermal CD123^+^ plasmacytoid dendritic cells, contributing to impaired local immune responses and increased susceptibility to inflammatory reactions [75].

The loss of skin barrier integrity makes DD patients more vulnerable to bacterial and viral infections. A microbiome analysis of scalp and chest lesions revealed a significant enrichment of Staphylococcus species compared to unaffected skin [76].

DD patients showed also increased susceptibility to herpes simplex virus (HSV) infection, leading to the occurrence of eczema herpeticum (EH). Severe EH can exacerbate skin disease by increasing serum levels of IL-6 and TNF-α, which downregulate the disease-causing gene *ATP2A2*, exacerbating the haploinsufficiency condition [77].

Together, these findings point out how SERCA2 dysfunction initiates a cascade of molecular and cellular impairment—spanning deregulated calcium signaling, ER stress, and immune dysregulation—leading to the complex pathophysiology of Darier’s disease.

## 3. Beyond the Skin: Exploring Systemic Manifestations

Since SERCA2b is expressed in all cell types and plays a crucial role in maintaining intracellular calcium balance, variants in *ATP2A2* associated with DD affect not only the skin but also other areas.

As already highlighted, oral and genital mucosae may be affected. Esophageal involvement has been described and confirmed histopathologically, but is considered extremely rare [78]. The present data are not sufficient to support screening with esophagogastroduodenoscopy, though oral and gastroesophageal signs and symptoms should be clinically evaluated at every visit.

Studies have shown that DD patients have an increased risk of developing metabolic disorders, neuropsychiatric abnormalities, and cardiac alterations, suggesting that DD should be considered a multi-organ disease.

Recent studies suggest a significant role of ER stress in the pathophysiology of both type 1 and type 2 diabetes. In pancreatic β cell, the intracellular calcium concentration is the primary regulator of insulin secretion [79]. In fact, recent reports highlight DD cases developing type I or type II diabetes as a comorbidity, potentially correlated with the pathophysiology of the disease. A population-based cohort study estimated a risk ratio of 1.74 (C.I. 1.13–2.69) for type 1 diabetes, while the correlation with type 2 was less significant [80].

The onset of type II diabetes could be explained by the failure of Ca^2+^ reuptake into the ER due to SERCA2b malfunctioning. Prolonged Ca^2+^ elevation leads to a sustained insulin secretion in response to hyperglycemia. However, excessive stimulation depletes insulin stores, contributing to the development of insulin resistance [60].

Additional evidence come from studies demonstrating that SERCA2 inhibition increases insulin secretion in a thapsigargin-induced manner [81] and triggers ER stress in pancreatic β cells, as indicated by the upregulation of ER stress markers, including CHOP, BIP, and XBP1 [60].

Intriguingly, in a non-obese diabetic (NOD) murine model prone to type I diabetes, the ER stress markers, such as BIP, XBP1s, and CHOP, were expressed, highlighting that ER stress activation may accelerate β cell death in the prediabetic phase of the disease. Another interesting aspect of this study was the detection in NOD mice of reduced SERCA2b protein expression in the ER [82]. The same phenomenon has been observed in patients with autoimmune diabetes [83].

Misfolded protein accumulation in the ER lumen can generate reactive oxygen species (ROS) [82]. β cells cannot tolerate high levels of ROS because they express low levels of ROS-detoxifying enzymes [84].

It has been suggested that skin inflammation, such as psoriasis, typical of DD patients, is closely related to an increased likelihood of developing type II diabetes [85]. ER stress induced by calcium imbalance may also affect the liver, where SERCA2b is the major isoform regulating lipid metabolism [86,87]. In summary, several studies are pointing towards an increased risk for diabetes, especially type 1, in DD patients, so a consultation with the endocrinologist/diabetologist may be needed in a small number of cases. Clinicians should be aware of symptoms like excessive thirst or hunger, frequent urination, unexplained weight loss, or fatigue.

The SERCA2b isoform is also highly expressed in neurons [88] which may account for the neurological manifestations observed in many DD patients, such as epilepsy, major depressive disorder, dysthymic disorder, bipolar I disorder, and dyslexia [29,30,34].

In the nervous system, as in the heart and skin, calcium released into the cytoplasm is pumped back into the ER by SERCA2. SERCA2b is ubiquitously expressed in neurons throughout the brain, with the highest levels in Purkinje cells, followed by hippocampal pyramidal cells and cortical levels II-V [89].

A recent study confirmed that SERCA2b is broadly distributed in neuronal subcellular fractions, with elevated levels in synaptic plasma membrane vesicles and microsome synaptosomes [90].

Due to the shared ectodermal origin, the skin and brain may show similar susceptibility to SERCA2 mutations [91], contributing to the high prevalence of neuropsychiatric comorbidities in DD patients.

In a 96-patient cohort, more than half of the individuals (55%) had been diagnosed with a psychiatric condition during their lifetime. Major depressive disorder was the most common (30%), followed by epilepsy (3%), bipolar disorder (4%), suicide attempts (13%), and suicidal thoughts (31%) [34].

A population-based study reported a potential genotype–phenotype correlation between *ATP2A2* variants and neuropsychiatric severity [92]. Specifically, variants predicted as loss-of-function variants, such as frameshift, splicing, nonsense, and start-loss variants, were associated with severe neuropsychiatric conditions such as bipolar disorder, schizophrenia, or affective psychosis. In addition, a combined literature-based analysis showed a significantly higher frequency of missense variants within the Stalk 4-TM4 (S4-M4) region in severe neuropsychiatric DD patients [23].

Another piece of evidence relies on the delay of cytosolic Ca^2+^ clearance after depolarization and on the enhancement of dopamine signaling, a common feature of schizophrenia and mood disorders [93].

Biochemical mechanisms link DD and neuropsychiatric phenotypes via the sphingosine–ceramide pathway and neuroinflammatory responses.

SERCA2b alterations lead to an imbalance of sphingosine–ceramide products which may induce neuron toxicity via an accumulation of ROS and cell death induction through the expression of the P2X7 death receptor (P2X7R), which may promote mood disorders via increased IL-1β and cortisol signaling [70,94].

Notably, sphingosine phosphate (S1P) plays a crucial role in keratinocyte adhesion and survival, thereby linking dyskeratotic and acantholytic skin phenotypes with neuropsychiatric features [95]. Neurons rely on calcium ions to regulate processes such as neurogenesis, neurotransmission, synaptic plasticity, and gene transcription. Indeed, it was observed that patients with Parkinson’s disease (PD) have higher average calcium levels than healthy individuals [96]. In PD patients, complexes formed by STIM1 and TRPC1 can inhibit CaV1.3, a voltage-gated calcium channel. This leads to the disruption of neuronal Ca^2+^ homeostasis and the development of PD symptoms. An excess of calcium levels also triggers mitochondrial stress, mitochondrial dysfunction, and even neuronal death [96]. Considering that SERCA2 dysfunction in Darier’s disease (DD) leads to intracellular calcium accumulation and TRPC1 overexpression [97], and that similar mechanisms of calcium dysregulation have been implicated in Parkinson’s disease (PD), it is plausible that these shared pathways contribute to DD patients’ increased risk of being diagnosed with PD [98].

Considering the high prevalence of neuropsychiatric symptoms, a neuropsychiatric and/or neurologic evaluation should be granted to every patient with DD, at least at the time of the diagnosis, with a follow-up to be determined based on the clinical features. Darier’s disease also appears to be associated with cardiac problems. In 2024 Frustaci and colleagues reported a rare case of a DD patient exhibiting a co-occurrence of cardiac symptoms, including arrhythmia and chest pain, as well as depressive syndrome. This patient was found to have a novel variant in the ATP2A2 gene, c.118G>A (p.Glu40Lys), classified as a VUS based on the ACMG criteria [31].

At the cardiac level, the SERCA2a isoform plays a crucial role in Ca^2+^ homeostasis by pumping calcium back into the sarcoplasmic reticulum (SR), thus causing cardiomyocyte relaxation. In heart failure (HF), it has been shown that SERCA2a expression is reduced, contributing to severe systolic and diastolic dysfunction [99,100,101]. By genetically disrupting SERCA2a expression, the HF phenotype is increased with reduced contractility, associated with ER stress [102].

Moreover, the SERCA2a haploinsufficiency in a DD mouse model led to hypertrophic cardiomyopathy, and when crossed with a transgenic model of increased myofibrillar Ca^2+^-sensitivity, it developed HF, suggesting that DD patients may be more susceptible to HF [103].

A cross-sectional case–control study involving 25 DD patients revealed that while these patients did not display HF markers, they showed specific, increased HF risk which was higher in DD women, supporting that DD is a multi-organ condition [104].

Even more recent evidence comes from a case report of a DD patient who began to experience clinical symptoms of muscle exhaustion, chest pain, and cardiac arrhythmias [31]. A cardiological evaluation with EKG should be considered at the time of the diagnosis, though more evidence is needed to support a full recommendation.

## 4. Treatments

Current therapies for DD can be divided into four main categories: oral retinoids, systemic therapies, topical treatments, and interventional procedures.

Retinoids, such as vitamin A derivatives (e.g., tretinoin and retinyl palmitate), regulate the hyperproliferation of keratinocytes. However, these treatments are associated with poor tolerability by DD patients [105]. Tazarotene (topical) and acitretin (oral), commonly used in the treatment of psoriasis, effective in the inflammatory forms, act on retinoic acid receptors and retinoid-X receptors by suppressing the proliferation of keratinocytes and the secretion of inflammatory cytokines. When retinoids are used in combination with other treatments, such as corticosteroids, methotrexate, and biologic drugs, the overall dose is reduced. Nevertheless, the side effects and teratogenic effects limit their use in clinical practice.

Alitretinoin, a vitamin A analogue binding both retinoic acid and retinoid-X receptors, exerts anti-inflammatory and immunomodulatory effects. Although the precise mechanisms are not fully understood, it reduces inflammation and improves the skin integrity with a reduced risk of teratogenicity. This allows for greater safety for women who wish to plan a pregnancy in the near future, unlike other retinoids that require longer suspension periods before conception [106].

Antibiotics, like doxycycline, are commonly used for secondary skin infections although they reduce inflammation, lead to epidermal hyperproliferation, and may modulate calcium release from the ER. It is important to note that it reaches therapeutic concentrations in most tissues throughout the body and easily crosses the blood–brain barrier. Gentamicin, via nonsense mechanism suppression, may promote the read-through of premature stop codons and thus have further therapeutic potential in DD [70].

Hypertrophic flexural skin lesions, such as in Darier’s disease, are often resistant to topical therapies.

Alternative therapeutic options are surgical and physical treatments, such as electrodessication, dermabrasion, CO_2_, and Erbium:YAG laser ablation, have resulted in significant lesion improvement and complete remission [105].

The main disadvantage of these non-selective techniques is that they may lead to permanent scarring.

The photodynamic technique (PDT), on the other hand, is more specific, stimulating a photo-sensitizing reaction only within the target tissue, minimizing the scarring risk, whilst inducing oxidative stress and cell apoptosis via the release of reactive oxygen species [107].

In severe and refractory DD patients, systemic monoclonal antibodies targeting the IL-23/IL-17 axis could be adopted. The administration of guselkumab, an anti-IL23A, or secukinumab, anti-IL17A, improved skin manifestations with reductions in itching, odor, and secondary infections within 1 year [73].

Recently, a 34-year-old Japanese woman with DD was successfully treated with calcipotriol/betamethasone dipropionate two-compound treatment [108]. Another recent study [109] focused on apremilast, orally administered to an 18-year-old female with DD with a missense *ATP2A2* variant. This phosphodiesterase 4 inhibitor was chosen based on the effectiveness in treating Hailey–Hailey disease (HHD), a genetically related disorder involving ATPases in epidermal calcium channels. Eight weeks post-initiation she exhibited significant improvements in the head, neck, and chest skin lesions.

A clinical trial concluded in 2021 employed botulin toxin injection and reported encouraging results, such as on itchiness, cutaneous pain, sweating and odor, infections, and psychosocial impairment; however, further studies are required to confirm the results on larger DD cohorts [110].

All of these therapeutic approaches, already tested on real patients, are the most promising ones for the near future and just need validation in wider cohorts.

Other repurposed drugs are under study, like Dantrolene, a muscle relaxer, may aid the retention of ER calcium, promote cell adhesion, reduce ER stress, and suppress apoptosis [111], but a solid in vivo demonstration is still lacking.

Among potential innovative therapies, mostly tested in vitro, recently the focus has shifted to sphingosin phosphate lyase (SGPL1), the canonical transient receptor potential 1 (TRPC1), and cyclooxygenase-2 (COX-2) [112]. The inhibition of SGPL1 compensates for the decrease in sphingosin kinase 1 (SPHK1), observed in DD patients (see neurological section), improving cell differentiation, E-cadherin assembly, and desmoplakin translocation [70]. The use of isotretinoin has been shown to reduce the influx of Ca^2+^ via TRPC1. TRPC1 overexpression has been suggested to lead to cell proliferation and apoptosis, which are responsible for the hyperkeratotic phenotype seen in Darier’s pathology [97]. Therefore, the therapeutic modulation of TRPC1 could be a potential treatment to alleviate keratinocyte phenotypes [70].

Moreover, an additional therapeutic strategy could be the blocking of the COX-2 enzyme, which has been shown to restore the expression of SERCA2 in keratinocytes [112].

Another therapeutic target could be the P2X7 receptor through an administration of naltrexone, which inhibits the opioid μ- and δ-receptors at the epidermis level, normalizing keratinocyte proliferation and adhesion [113].

In summary, common treatments for DD still rely on symptom management and behavioral modifications to avoid triggers. The most used therapeutic measures are retinoids (with or without corticosteroids), antibiotics, photodynamic therapy, and surgical excision. There is not yet a validated targeted treatment for the underlying pathogenesis. Drug repurposing can be a solution in some cases, but there are no shared guidelines yet. Understanding the molecular mechanisms behind DD is essential to identify new therapeutic targets. This could allow the development of innovative mechanism-based approaches capable not only of reducing the symptoms but also addressing the pathologic cause.

## 5. Conclusions and Future Perspectives

In conclusion, the comprehensive understanding of Darier’s Disease (DD) necessitates addressing several critical aspects to improve patient care and therapeutic outcomes:

- Multisystemic nature: DD, an autosomal dominant acantholytic dermatosis, affects skin and mucous membranes, and extends beyond dermatological manifestations to involve neurological, cardiovascular, and metabolic systems. A multi-systemic approach is essential for evaluating patient management and a comprehensive assessment of dermatological, neurological, and cardiac symptoms is crucial. Thereby, a multidisciplinary approach, involving dermatologists, neurologists, cardiologists, and geneticists, is necessary for comprehensive DD management and improved patient outcomes.
- Need for longitudinal studies: Further research, including longitudinal clinical studies and multi-omics analyses, will be crucial to unravel the systemic implications of DD and its comorbidities.
- Unresolved mechanisms: Key gaps remain in understanding the interplay between ER stress, inflammatory pathways, and disease pathogenesis, which could reveal novel therapeutic targets. Moreover, skin microbial imbalances and immune dysfunction studies may open doors to innovative treatments, such as immunomodulatory or microbiome-targeted therapies.
- Symptom-based therapy: The therapeutic approach still relies on retinoids, photodynamic therapy, and surgical excision for the management of skin manifestations, but more targeted treatments are under study.
- Patient-centered focus: Applying personalized medicine and addressing psychosocial impacts, such as quality of life and mental health, should be integral to DD care, given its chronic and stigmatizing nature.

## 6. Methods

This study is a narrative review aimed at summarizing and contextualizing the current literature on the genetic, clinical, and systemic manifestations of Darier’s disease, with a particular focus on associated comorbidities and potential therapeutic approaches.

### 6.1. Literature Search and Selection

A systematic literature search was conducted using the PubMed/MEDLINE database up to the year 2025. The following keywords were used in various combinations: “Darier disease”, “ATP2A2”, “Darier disease AND mutations”, “SERCA2b dysfunction”, “Darier AND neurological manifestations”, “Darier disease AND psoriasis”, “Darier AND cardiac disease”, and “Darier disease treatment”. No language, regional, or temporal restrictions were applied. The search was limited to specific article types, including clinical trials, randomized controlled trials, and reviews indexed in PubMed.

After removing duplicates, titles and abstracts were screened for relevance. Full texts were reviewed to confirm eligibility. A total of 112 articles were selected based on their relevance to the research questions and methodological quality.

### 6.2. Inclusion/Exclusion Criteria and Data Extraction

Eligible studies included original, peer-reviewed articles focused on human subjects and experimental models of Darier’s disease (including mouse and pig models). Case reports discussing additional clinical features (e.g., cardiac, neurological, and metabolic manifestations) associated with DD were also included. Exclusion criteria were conference abstracts, editorials, reviews, and non-peer-reviewed sources.

## Figures and Tables

**Table 1:** Clinical and genetic findings in DD associated with the most frequent *ATP2A2* variants. Thirty variants were identified in 65 patients, affecting 17 distinct amino acid positions in the ATP2A2 gene sequence. For each variant, the total number of patients in whom it was identified, without considering family history data, is reported, except for the c.530A>C (p.Gln177Pro) variant, which is discussed in greater detail in the text. The complete list of all patients included in the study is provided in Supplementary Table S1 and all recurrency numbers are shown in Figure 3. N.a.: not available, -: absence of the clinical characteristic, and NOS: not otherwise specified. ACMG Classification of Genetic Variants: Pathogenic (P), Likely Pathogenic (LP), and Variant of Uncertain Significance (VUS).

Involved Amino Acid Position (n = 17)	Patients (n = 62)	Variants (n = 30)	Type	Protein Domain	ACMG Classification (P/LP/VUS)	Severity of DD	Skin Phenotype	Neuropsychiatric Disorder	Cardiac Defects	Ref.
p.Met1	n = 4	c.1A>G p.Met1Val	Missense	Start codon	P	Mild/Moderate	Hyperkeratotic patches on abdomen.	Major depressive/Bipolar disorder.	N.a.	[23,30,32]
	n = 1	c.1A>T p.Met1Leu	Missense	Start codon	P	N.a.	N.a.	Bipolar disorder.	N.a.	[30]
p.Gly23	n = 2	c.68G>A p.Gly23Glu	Missense	S1	P	N.a.	N.a.	N.a.	N.a.	[26,30]
c.118 (splice site)	n = 1	c.118+3A>T	Splice site	S1	VUS	Severe	Excoriated papules neck and seborrheic areas trunk, extensive hyperkeratotic flexural patches, and dystrophic toe nails.	-	-	[30]
	n = 1	c.118G>A p.Glu40Lys	Splice site	S1	VUS	Severe	Extensive greasy, crusted, yellow–brown papules on the seborrheic areas.	Depressive syndrome with a failed attempt of suicide.	Palpitations and chest pain at rest.	[31]
p.Arg131	n = 4	c.392G>A p.Arg131Gln	Missense	A domain	P	Mild/Moderate	Extensive confluated verrucous plaques in seborrheic areas, V-shaped notches.	N.a.	-	[24,30,32]
	n = 3	c.391C>T p.Arg131*	Nonsense	A domain	LP	Moderate/Severe	Hyperkeratotic lesions on the upper trunk and scalp with severe exacerbation during summer.	-	N.a.	[25]
p.Pro160	n = 2	c.479C>T p.Pro160Leu	Missense	A domain	LP	Severe	Disseminated hyperkeratotic papules and confluating patches on trunk and extremities.	Learning disability and depressive symptoms.	-	[29,30]
p.Gln177	n = 7	c.530A>C p.Gln177Pro	Missense	A domain	LP	Moderate	Few skin and oral lesions. Warty, greasy, hyperkeratotic papules and plaques over the scalp, upper trunk, and forearms. Large subcutaneous cysts over his face. Numerous hyperkeratotic papules on the trunk, subungual hyperkeratosis, and few oral lesions. Nail changes and few skin lesions located on the upper chest and flexures.	Depression NOS.	ECG abnormalities (prolonged QT interval).	[25]
p.Thr317	n = 1	c.948del p.Thr317Profs*68	Frameshift	S4	LP	Moderate	N.a.	Psychiatric disorder NOS. Suicide attempt. Epilepsy.	N.a.	[23]
	n = 1	c.949_956del p.Thr317Glyfs*56	Frameshift	S4	LP	Moderate	N.a.	Major depressive disorder. Suicide attempt. Investigations for a blackout.	N.a.	[23]
p.Ile348	n = 2	c.1043T>C p.Ile348Thr	Missense	P domain	P	Moderate/Severe	Recurrent infections and eczema-like perioral lesions.	Depression.	N.a.	[23,29]
p.Thr357	n = 2	c.1070C>A p.Thr357Lys	Missense	P domain	LP	Moderate	N.a.	Major depressive disorder. Investigations for hearing problems.	N.a.	[23,26]
	n = 1	c.1070C>G p.Thr357Arg	Missense	P domain	LP	Mild	N.a.	-	N.a.	[23]
	n = 1	c.1070_1082del p.Thr357Serfs*24	Frameshift	P domain	LP	Mild	Hyperkeratotic papules in seborrheic areas.	-	-	[30]
p.Ser495	n = 3	c.1484C>T p.Ser495Leu	Missense	N domain	LP	Moderate	N.a.	Major depressive disorder.	N.a.	[23,26]
p.Arg673	n = 2	c.2017del p.Arg673Alafs*15	Frameshift	P domain	LP	Moderate	N.a.	Major depressive disorder. Investigations for fainting episodes.	N.a.	[23,26]
p.Lys683	n = 2	c.2047A>G p.Lys683Glu	Missense	P domain	LP	Mild–Moderate	N.a.	N.a.	N.a.	[29,30]
	n = 1	c.2046insC p.Lys683Glnfs*3	Frameshift	P domain	LP	Moderate	N.a.	Anxiety disorder NOS.	N.a.	[23]
	n = 1	c.2048A>T p.Lys683Met	Missense	P domain	LP	Moderate	N.a.	Investigations for blackouts.	N.a.	[23]
	n = 1	c.2048A>G p.Lys683Arg	Missense	P domain	LP	Mild–Moderate	Classical distribution	N.a.	-	[29]
p.Ser765	n = 3	c.2294C>T p.Ser765Leu	Missense	M5	LP	Mild/Moderate	Lower back, knee folds, and waist.		N.a.	[23,33]
	n = 1	c.2294C>G p.Ser765Trp	Missense	M5	LP	Mild	Hyperkeratotic papules in seborrheic areas.	Dyslexia and mild apraxia.	-	[30]
p.Asn767	n = 5	c.2300A>G p.Asn767Ser	Missense	M5	P	Mild/Moderate	N.a.	Major depressive disorder. Treatment for hearing problems. Investigations for poor memory.	N.a.	[23,32]
	n = 1	c.2299A>G p.Asn767Asp	Missense	M5	P	Mild–Moderate	Eruptions on scalp, neck, trunk, limbs, and nails.	-	N.a.	[28]
p.Asn795	n = 2	c.2384A>G p.Asn795Ser	Missense	M6	VUS	Moderate	Widespread hyperkeratotic papules incl dorsum of hands, longitudinal leukonychia, and palmar pits.	Anxiety disorder NOS.	N.a.	[23,30]
	n = 1	c.2385T>G p.Asn795Lys	Missense	M6	VUS	Severe	Entire body and pharynx.	N.a.	N.a.	[33]
p.Tyr894	n = 3	c.2678dup p.Tyr894Ilefs*17	Frameshift	M7-M8	LP	Mild	N.a.	Major depressive disorder.	N.a.	[23,32]
p.Ser920	n = 1	c.2759C>T p.Ser920Phe	Missense	M8–M9	LP	Moderate	N.a.	Depression NOS.	N.a.	[23]
	n = 2	c.2759C>A p.Ser920Tyr	Missense	M8–M9	LP	Severe	N.a.	Investigations for one-sided weakness. Medical notes report “adjustment reaction” to relapse in DD.	N.a.	[23]

## Data Availability

All relevant data are available from the corresponding author upon request.

## Supplementary Materials

The following supporting information can be downloaded at https://www.mdpi.com/article/10.3390/genes16060619/s1. Supplementary Table S1. Clinical and genetic findings in literature-based reported cases with *ATP2A2* variants. A total of 189 patients found in the literature exhibited 143 *ATP2A2* variants and showed a heterogenous clinical phenotype. N.a.: not available, +: presence of the clinical feature, -: absence of the clinical characteristic, NOS: not otherwise specified, and AKV: Acrokeratosis verruciformis of Hopf. ACMG Classification of Genetic Variants: Pathogenic (P), Likely Pathogenic (LP), and Variant of Uncertain Significance (VUS).
